# Unveiling Optimal Synthesis and Structural Insights of Starch Ferulate via the Mechanoenzymatic Method

**DOI:** 10.3390/foods12203715

**Published:** 2023-10-10

**Authors:** Jingxue Liu, Tingting Gao, Jiaying Xin, Chungu Xia

**Affiliations:** 1 Key Laboratory for Food Science and Engineering, Harbin University of Commerce, Harbin 150028, China; 2 College of Food Engineering, Jilin Agricultural Science and Technology University, Jilin 132101, China; 3 State Key Laboratory for Oxo Synthesis & Selective Oxidation, Lanzhou Institute of Chemical Physics, Chinese Academy of Sciences, Lanzhou 730000, China

**Keywords:** starch ferulate, mechanoenzymatic synthesis, twin screw extrusion, lipase catalysis, modified corn starch

## Abstract

In this study, starch ferulate was synthesized employing a mechanoenzymatic method, specifically based on the twin screw extrusion technique and lipase catalysis. The research then primarily centered on optimizing process parameters and conducting structural analysis. Optimal conditions were determined to be 8.2% ferulic acid addition, 66 °C extrusion temperature, and 3.2% lipase (N435) addition. The enzyme-catalyzed time was 30 s. The degree of substitution for starch ferulate was quantified at 0.005581 under these specific conditions. The presence of C=O bonds in the synthesized starch ferulate proved that the synthesis process was efficient. Additionally, the crystal structure underwent reconstruction. Observations through Scanning Electron Microscopy (SEM) and Confocal Laser Scanning Microscopy (CLSM) demonstrated that the mechanoenzymatic method led to an augmentation in the specific surface area of starch molecules, thereby facilitating the exposure of active sites. This breakthrough underscores the vast potential of mechanoenzymatic techniques to revolutionize the rapid and sustainable synthesis of starch ferulate, marking a pioneering stride in ester synthesis. The insights garnered from this study transcend theory, offering a visionary roadmap for the development and real-world deployment of advanced modified starch esters.

## 1. Introduction

Starch-based materials are now often used to meet individualized nutritional needs. Their unique physicochemical characteristics have also been shown to facilitate the precise delivery and release of active substances [1]. Corn starch (CS), a readily available and cost-effective biopolymer, is a dual-helical polymer with the capacity for environmentally friendly degradation [2]. It possesses a sweet taste and neutral property, along with demonstrated effects in reducing blood pressure and cholesterol levels. Corn starch, rich in a spectrum of nutritional and wellness constituents, notably includes glutathione, known for its anticancer properties. Its content of tocopherols and linoleic acid contributes to the reduction in cholesterol levels within the body, consequently mitigating the progression of arterial sclerosis. Additionally, the trace element selenium, present in corn starch, facilitates the breakdown of oxidative compounds within the body, thus exerting a tumor-inhibiting effect [3]. Ferulic acid (FA), a phenolic acid derivative of cinnamic acid, is widely distributed in nature. Numerous traditional Chinese medicinal herbs, plants, and grains serve as natural sources of ferulic acid, including Angelica sinensis, Ligusticum chuanxiong, rice bran, and various plant seeds. Functioning as a versatile bioactive compound in traditional medicine and several plant species, ferulic acid finds extensive application in contemporary pharmaceutical and pathological research [4]. Characterized by its lack of odor and mild bitterness, ferulic acid exhibits solubility in hot water, ethanol, and ethyl acetate, while remaining insoluble in benzene, ether, and petroleum ether.

In recent years, within the food and beverage industries, ferulic acid has frequently been incorporated as a health-promoting factor into various products, thereby enhancing their functional benefits [5]. Nevertheless, the administration of ferulic acid via oral or injectable routes presents challenges in achieving targeted delivery to the colon for optimal therapeutic efficacy. The colonic release of ferulic acid is contingent upon its sequestration by dietary fibers. Moreover, its involvement in hepatic–enteric circulation is notably constrained, mandating the catalytic activity of esterases produced by the colonic microbiota for the liberation of ferulic acid [6]. Additionally, due to its challenging direct reactivity with starch, ferulic acid is employed as the guest molecule, while starch serves as the main carrier molecule [7]. Through a catalytic process involving lipase, they undergo esterification, resulting in the formation of ferulic acid starch esters.

The synthesis of starch ferulate (SF) effectively addresses the challenge of ferulic acid’s limited ability to reach the colon for functional impact. Starch ferulate, as an innovative variant of modified starch (esterified starch), demonstrates a diverse array of functionalities encompassing anti-depressant, hypoglycemic, anti-colorectal cancer, cardiovascular disease treatment, antioxidant, and thrombosis regulation properties [8]. Furthermore, starch ferulate significantly enhances the physical properties of starch by reducing its brittleness and increasing film-forming characteristics. This modification plays a crucial role in inhibiting starch aging, enzymatic degradation, and digestive absorption efficiency [9].

In view of the advantage that mechanoenzymatic catalysis possessed in terms of high efficiency and lack of dependence on organic solvents, we have undertaken the utilization of twin screw extrusion (TSE) technology to synthesize starch ferulate. Twin screw extrusion employs mechanical shear forces, high-frequency vibrations, and frictional forces under high-temperature and high-pressure conditions to induce physical and chemical changes in materials [2,10]. As an example, starch molecules have the capacity to shift from ordered to disordered states, thereby disrupting starch crystalline structures and modifying the surface characteristics of starch particles. This transformation exposes a multitude of reactive sites on starch molecules. With the catalytic action of lipase, ferulic acid is facilitated to penetrate into the cavities created by the breakdown of the starch structure. This interaction maximizes the contact between ferulic acid and starch molecules. Extrusion processing offers notable advantages, including operational simplicity, cost-effectiveness, low resource utilization, and environmental compatibility, rendering it a versatile and widely applicable technique within the domain of food processing [11].

Corn starch, as a resistant starch, also has excellent physiological activity and immunomodulatory potential. Therefore, its further modification and application is essential [12]. This study focuses on corn starch and ferulic acid as the primary substrate materials. Utilizing a twin screw enzymatic extrusion process, ferulic acid corn starch ester was synthesized, resulting in an innovative composite process that is environmentally friendly and operationally straightforward. This process effectively enhances the degree of substitution of ferulic acid corn starch ester. Structural characterization and analysis of the extrusion-modified ferulic acid starch ester have been conducted, providing insights that contribute to the comprehensive development and utilization of corn starch and ferulic acid. This research provides a valuable reference for broadening the potential applications of ferulic acid starch esters.

## 2. Materials and Methods

### 2.1. Materials

Corn starch (food grade) was sourced from China National Cereals, Oils and Foodstuffs Corporation (COFCO) Biochemical & Energy Co., Ltd. (Changchun, China). Ferulic acid (99%) was purchased from Aladdin Biochemical Technology Co., Ltd. (Shanghai, China). Lipase (N435, 100,000 U/g) was supplied by Bide Pharmaceutical Technology Co., Ltd. (Shanghai, China). The rest of the reagents (analytically pure) were provided by Sinopharm (Beijing, China).

### 2.2. Preparation of Starch Ferulate

Corn starch was mixed with water (20%, *w*/*w*), and this starch solution was combined with the iso-octane solution (20 mL) of lipase. Upon the addition of ferulic acid, the mixture was processed using a twin screw extruder (SLG-30-ZV, SiBiono, Shenzhen, China) at a speed of 20 Hz [13]. The enzyme-catalyzed time was 30 s. The experimental parameters were systematically varied, considering lipase dosage, ferulic acid dosage, and extrusion temperature as distinct factors. Following the extrusion process, the resulting material was subjected to a drying step followed by comminution. Subsequently, it underwent a series of purification steps involving washing with anhydrous ethanol via centrifugation. The supernatant obtained from each purification cycle was meticulously examined employing thin-layer chromatography and UV spectroscopy to unequivocally confirm the absence of unbound ferulic acid. This iterative purification process was repeated until no residual free ferulic acid was detectable. Ultimately, the resulting precipitate was subjected to a final drying step in preparation for subsequent utilization.

### 2.3. Single-Factor Experiment

#### 2.3.1. Effect of Ferulic Acid Addition on the Degree of Substitution

Under the conditions of a screw rotation speed of 20 Hz, a water addition rate of 20% (*w*/*w*), 3% lipase dosage, and an extrusion temperature of 65 °C, the evaluation was carried out to assess the influence of different levels of ferulic acid (4%, 6%, 8%, 10%, and 12%) on the degree of substitution.

#### 2.3.2. Effect of Extrusion Temperature on the Degree of Substitution

Under the conditions of a screw rotation speed of 20 Hz, a water addition rate of 20% (*w*/*w*), ferulic acid dosage of 8%, and lipase dosage of 3%, the evaluation was performed to analyze the impact of different extrusion temperatures (55 °C, 60 °C, 65 °C, 70 °C, and 75 °C) on the degree of substitution.

#### 2.3.3. Effect of Lipase Addition on the Degree of Substitution

Under the conditions of a screw rotation speed of 20 Hz, a water addition rate of 20% (*w*/*w*), ferulic acid dosage of 8%, and an extrusion temperature of 65 °C, the assessment was conducted to analyze the influence of different levels of lipase dosage (1%, 2%, 3%, 4%, and 5%) on the degree of substitution.

### 2.4. Response Surface Test

Using ferulic acid dosage (A), extrusion temperature (B), and lipase dosage (C) as individual factors, and degree of substitution (Y) as the response value, a three-factor three-level response surface experiment was designed. It should be noted that since the degree of substitution was a measure of the degree of modification, it was used as a criterion for response surface optimization. The Design-Expert software (v 8.0.6) and the principles of Box–Behnken design were employed to determine the optimal modification process parameters for preparing starch ferulate using twin screw extrusion.

### 2.5. Degree of Substitution Determination

The degree of substitution of starch ferulate was determined by a TU-1950 UV-Vis spectrophotometer (Purkinje GENERAL Instrument Co., Ltd., Beijing, China). Following the extraction of the hydrolyzed starch ferulate solution using ethyl acetate, the absorbance value of alkali-dissolved ferulic acid was determined. Subsequently, the degree of substitution was calculated using a linear regression equation to ascertain the concentration of ferulic acid [8].

### 2.6. Plotting of Standard Curves

**Figure 1:**
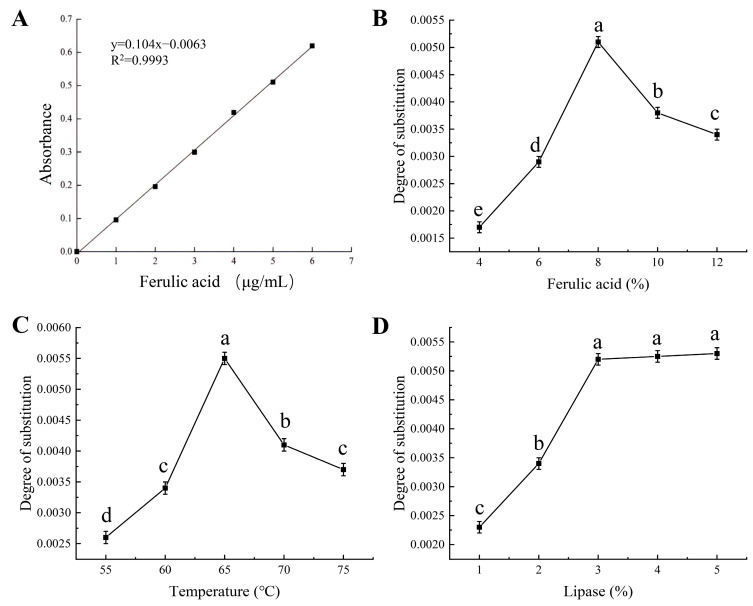
(**A**) Ferulic acid concentration–absorbance standard curves and the effects of (**B**) ferulic acid, (**C**) temperature and (**D**) lipase on degree of substitution. Different lowercase letters represent significant differences (*p* < 0.05).

Standard solutions of ferulic acid were prepared with mass concentrations of 0, 1, 2, 3, 4, 5, and 6 μg/mL. The absorbance values of these solutions were subsequently measured at 320 nm. Using the absorbance values as the vertical coordinates and the mass concentrations of ferulic acid solutions as the horizontal coordinates, standard curves were plotted (Figure 1A). The linear regression equation is y = 0.104 × −0.0063, R^2^ = 0.9993.

### 2.7. Hydrolysis of Starch Ferulate

A mass of 0.10 g of starch ferulate was placed within a conical flask and combined with a 1 mol/L NaOH solution. Stirring was conducted magnetically at 50 °C for a duration of 3 h to induce hydrolysis. Subsequently, the pH was adjusted to 2 with a 1 mol/L HCl solution while maintaining the elevated temperature. After a defined time interval, the hydrolyzed solution was subjected to extraction using ethyl acetate, a procedure that was repeated four times. The resulting extracted solution was set aside for subsequent utilization.

### 2.8. Calculation of Degree of Substitution

The absorbance value of the extract was measured at 320 nm and the ferulic acid content in the starch ferulate was calculated from the standard curve. The degree of substitution was calculated according to the following equations [14].

(1) $$W=\frac{C\times V}{m_{0}}\times 100$$

(2) $$DS=\frac{162W}{100M-\left(M-1\right)\times W}$$

where *W* (%) is the mass fraction of ferulic acid in starch ferulate; *C* (mg/L) is the mass concentration of ferulic acid calculated from the standard curve; *V* (mL) is the volume of the extraction solution; *m*_0_ (g) is the mass of starch ferulate; *M* (194.19) is the relative molecular mass of ferulic acid; 162 (g/mol) is the molar mass of one glucose unit; and *DS* is the amylose substitution degree.

### 2.9. Determination of Free Ferulic Acid in Reaction Products

Before calculating the degree of substitution, it is essential to perform a washing step on the reaction product obtained after twin screw extrusion. This washing process involves centrifugation with anhydrous ethanol, followed by the analysis of the supernatant using thin-layer chromatography (TLC). Specifically, an appropriate amount of unfolding agent (hexane: ether: benzene: dichloromethane = 1: 10: 15: 25) was pipetted into the unfolding cylinder. The centrifugation supernatant was then dipped into a spotting capillary and spotted on a silica gel thin-layer plate. And ferulic acid standard was utilized as a control at the same horizontal position. Once the silica gel thin-layer plate had been impregnated and penetrated by the elution solvent to a distance of 3 cm from the upper edge, it was carefully extracted and subjected to air-drying. Subsequently, the silicone thin-layer plate was exposed to ultraviolet lamp irradiation. If only a singular discernible fluorescent sample spot manifested, it signified successful purification, and the plate was deemed clean and ready for the final drying process, preparing it for subsequent applications.

### 2.10. Detection of Free Ferulic Acid by UV Spectroscopy

The samples were scanned by UV full wavelength (200–800 nm) spectroscopy (Purkinje GENERAL Instrument Co., Ltd., Beijing, China) while anhydrous ethanol was used as a control [15].

### 2.11. Fourier Transform Infrared Spectroscopy (FTIR) Analysis

A total of 0.001 g of each sample (comprising corn starch, extruded corn starch, ferulic acid, and starch ferulate) was individually weighed alongside 0.1 g of dried KBr. These components were subsequently placed within an onyx mortar and meticulously ground, maintaining a sample to KBr ratio of 1:100. The resulting mixture was then compressed using a tablet press at 20 MPa for a duration of 5 min. The pressed sample tablet was placed into the infrared detector, and the pure KBr tablet was used as a blank [16]. Scanning range: 400–4000 cm^−1^, resolution: 4 cm^−1^, number of scans: 32 times.

### 2.12. X-ray Diffraction (XRD) Analysis

A 2 g sample was pressed and analyzed with an X-ray diffractometer using Cu-Kα rays as an excitation source. The scanning range was 5°~50° (2θ) and the scanning rate was 4 (°)/min [17].

### 2.13. Scanning Electron Microscope (SEM) Analysis

Double-sided conductive adhesive was securely attached to the sample stage, and a cotton swab was immersed in the sample powder, subsequently affixed to the conductive adhesive. Following this, the sample stage was positioned within the ion sputtering instrument for the purpose of vacuum-coating with gold. This procedure aimed to enhance the conductivity of the sample, thereby facilitating the enhancement of resolution [18]. Probe: secondary electron (SE2); accelerating voltage: 15 kV; magnification of 1000 times and 2000 times.

### 2.14. Confocal Laser Scanning Microscopy (CLSM) Analysis

The samples (4 mg) were mixed with 15 μL of FITC acetone solution (0.1 mg/mL) protected from light. After 20 min, the suspension was dropped on a slide and observed at an excitation wavelength of 498 nm and an emission wavelength of 517 nm [19].

### 2.15. Data Statistics and Analysis

All experiments were conducted in triplicate. The results are expressed as the mean ± standard deviation and were statistically analyzed for significance using SPSS software at the significance levels of *p* < 0.01 or 0.05.

## 3. Results and Discussion

### 3.1. Single-Factor Experiment

#### 3.1.1. Effect of Ferulic Acid Addition on Degree of Substitution

It should be noted at the outset that due to the high cost of ferulic acid and lipase as well as the high energy consumption due to heating, we prioritized the effects of ferulic acid, lipase and temperature on the degree of substitution.

The right amount of ferulic acid had an essential effect on the esterification reaction. According to the principle of chemical reaction equilibrium, increasing the amount of ferulic acid promoted the forward rate of the reaction, thus increasing the amount of product generation [20]. Excessive amounts of ferulic acid, however, tend to hinder the effective collision of molecules. As depicted in Figure 1B, the degree of substitution increased from 0.0017 to 0.0051 as the ferulic acid addition was elevated from 4% to 8%. This was due to the fact that at lower concentrations, ferulic acid entered into the molecule through the disrupted apertures of the corn starch and bound to the hydroxyl group of the starch, and the rate of esterification in the reactive site increased, which led to the increase in the degree of substitution [21]. When the quantity of ferulic acid added exceeded 8%, a reduction in the degree of substitution was observed, declining from 0.0051 to 0.0034. This phenomenon could be attributed to several factors. Firstly, it suggested that the addition of ferulic acid approached a saturation point, with an insufficient quantity of available hydroxyl groups on the corn starch molecules available for substitution [22]. On the other hand, an excessive amount of ferulic acid would hinder the effective collision among molecules, leading to a rapid increase in the viscosity of the reaction system and a rapid increase in the temperature of the material thereby degrading the starch and decreasing the degree of substitution [14]. Thus, 8% was chosen as the optimal addition amount of ferulic acid.

#### 3.1.2. Effect of Extrusion Temperature on Degree of Substitution

Suitable extrusion temperature was favorable for the esterification reaction and could accelerate the pasteurization of corn starch and the increase in active sites. Nevertheless, excessively high extrusion temperatures can result in rapid volume expansion and molecular degradation of corn starch, thereby adversely impacting the esterification reaction. As illustrated in Figure 1C, the degree of substitution increased from 0.0026 to 0.0055 as the extrusion temperature was raised from 55 °C to 65 °C. When the extrusion temperature was increased in this range, the viscosity of the reaction system decreased and the rate of intermolecular thermal movement increased, which led to the enhancement of the effective collision among the reactive substrate and enzyme molecules, and accelerated the esterification reaction [23]. Moreover, lipase had high stability in this temperature range and could give full play to its vitality. Also, the increase in temperature accelerated the destruction of starch crystal structure and the unfolding of helical structure, which was beneficial to straight-chain starch to expose more reactive active sites [11]. Nonetheless, when the extrusion temperature exceeded 65 °C, two detrimental effects were observed. Firstly, the elevated temperature led to starch expansion, resulting in the degradation of corn starch into oligosaccharides. Secondly, the increased heat had an adverse impact on the activity of lipase, causing a loss of enzymatic function and thereby hindering the progression of the esterification reaction. Consequently, the optimal extrusion temperature was determined to be 65 °C, striking a delicate balance that allowed for efficient esterification while minimizing starch degradation.

#### 3.1.3. Effect of Lipase Addition on Degree of Substitution

The concentration of lipase played a pivotal role in the esterification reaction involving corn starch, significantly influencing the catalytic efficiency of the entire reaction system. Inadequate lipase concentration failed to ensure the completion of the esterification reaction, while an excessively high lipase concentration led to unwarranted wastage, primarily due to the saturation of the esterification process. As shown in Figure 1D, the degree of substitution was the smallest at a lipase addition of 1%, with a value of 0.0023. The degree of substitution increased with the increase in lipase concentration, which could be attributed to the disruption of the surface structure of the extruded starch granules, and the exposure of the reactive sites provided favorable conditions for catalysis [24]. When the lipase addition was increased to 3%, the degree of substitution was 0.0052. However, as the lipase concentration continued to increase, the rise in the degree of substitution became less pronounced. This phenomenon can be attributed to the fact that an excessive lipase addition limited the number of reactive active sites exposed by the starch granules, resulting in a smaller increase in the degree of substitution [25]. Consequently, the optimum lipase addition was 3%.

### 3.2. Box–Behnken Experimental Design and Analysis

After multiple regression fitting, ANOVA, and significance testing of Table 1, a quadratic regression equation with degree of substitution as the objective function was derived:

Y = 5.468 × 10^−3^ + 2.564 × 10^−4^A + 5.133 × 10^−4^B + 2.746 × 10^−4^C − 7.850 × 10^−5^AB − 3.028 × 10^−4^AC + 2.520 × 10^−4^BC − 8.588 × 10^−4^A^2^ − 9.151 × 10^−4^B^2^ − 9.733 × 10^−4^C^2^. (3)

A significance test for this model yielded the results of the ANOVA in Table 2 and the results of the regression model credibility analysis in Table 3.

As shown in Table 2 and Table 3, the regression model had *R*^2^ = 0.9923 with *p*-value < 0.0001, indicating that the regression model was highly significant. And the Lack of fit was not significant (*p*-value > 0.05), indicating that the predicted values of the regression model fit the actual values with high reliability. From the F-value, it could be seen that the effect of each factor on the degree of substitution was extrusion temperature (B) > lipase addition (C) > ferulic acid addition (A).

### 3.3. Effect of Interaction of Factors on Degree of Substitution

**Figure 2:**
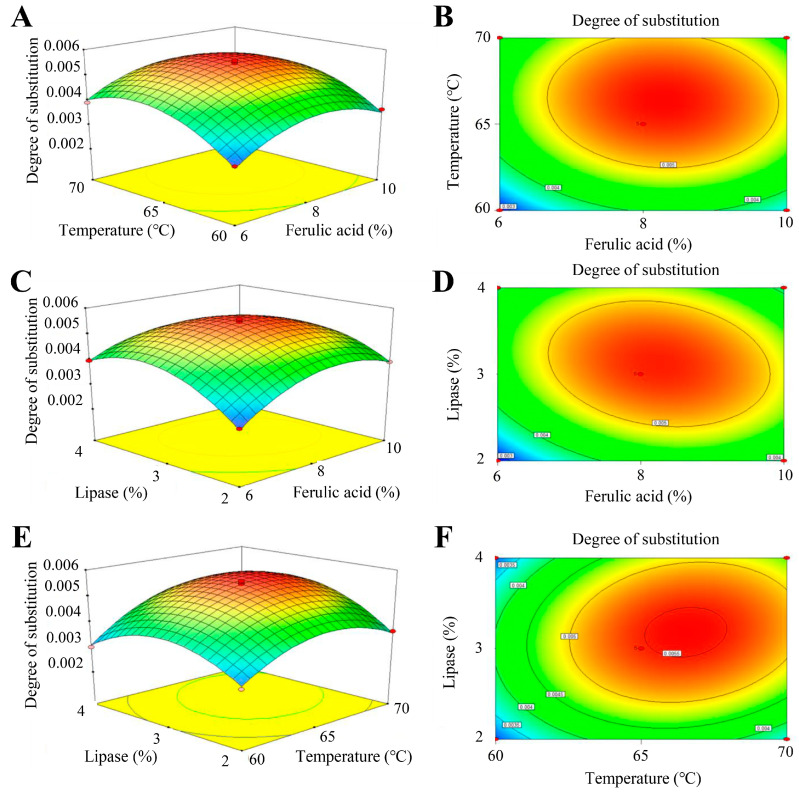
(**A**,**B**) Interactions between ferulic acid content and temperature, (**C**,**D**) between ferulic acid content and lipase content, (**E**,**F**) and between temperature and lipase content on degree of substitution.

From Figure 2, it could be seen that the interaction between ferulic acid (A) and lipase (C) was highly significant; the interaction between extrusion temperature (B) and lipase (C) was highly significant; and the interaction between ferulic acid (A) and extrusion temperature (B) was not significant.

### 3.4. Optimization of Process Parameters

In order to determine the optimal process parameters, the triple quadratic equations were derived by calculating the first partial derivatives of the fitted regression equations, respectively:

2.564 × 10^−4^ − 7.850 × 10^−5^B − 3.028 × 10^−4^C − 8.588 × 10^−4^A = 0 (4)

5.133 × 10^−4^ − 7.850 × 10^−5^A + 2.520 × 10^−4^C − 9.151 × 10^−4^B = 0 (5)

2.746 × 10^−4^ − 3.028 × 10^−4^A + 2.520 × 10^−4^B − 9.733 × 10^−4^C = 0 (6)

The optimal process parameters were 8.21% ferulic acid addition (A), 66.49 °C extrusion temperature (B) and 3.16% lipase addition (C). Under these conditions, the degree of substitution was 0.005581. Considering the realistic production, the optimal factor conditions were adjusted to 8.2% ferulic acid addition, 66 °C extrusion temperature and 3.2% lipase addition. After three parallel experiments, the degree of substitution was 0.005581.

Based on the aforementioned discoveries, it is evident that the mechanoenzymatic synthesis of starch esters presents significant advantages. The process was characterized by eco-friendly attributes, avoiding the need for organic solvents and elevated temperatures. High selectivity was achieved, minimizing unwanted side reactions. Remarkably controlled under mild conditions, the method exhibited strong adaptability to diverse substrates. Limited catalyst usage reduced costs and waste. The feasibility of precise control over reactions was highlighted, achieved through strategic adjustments of parameters. Furthermore, the scope of application extended across a diverse range of substrates, highlighting its extensive versatility. This methodology serves as an exemplar of the synergy between environmental sustainability, controlled synthesis, and adaptability, thus propelling the development of sustainable and effective avenues within the realm of ester synthesis.

### 3.5. TLC Determination

**Figure 3:**
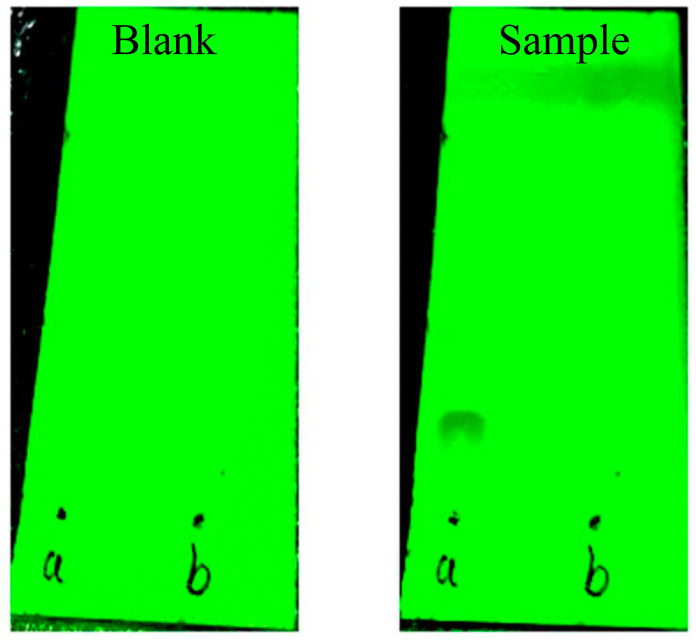
TLC results of residual free ferulic acid in the reaction products.

As shown in Figure 3, on the sample silica gel plate, ferulic acid was clearly seen to be isolated above the sample point a, while it was not observed above the sample point b, which proved that the residual free ferulic acid in the reaction product was washed by anhydrous ethanol.

### 3.6. UV Spectral Determination

**Figure 4:**
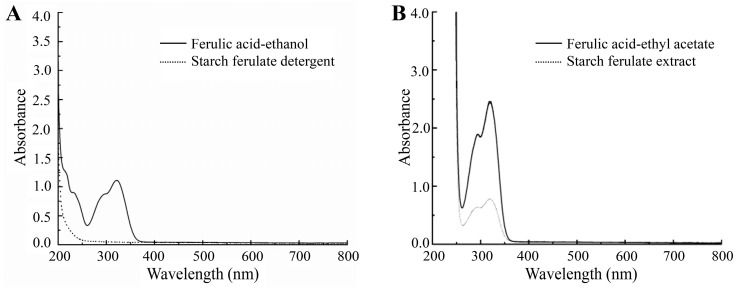
(**A**) Determination of ferulic acid in the detergent solution of the product. (**B**) Determination of ferulic acid in the hydrolysate of the product.

The detergent of the product was subjected to UV full wavelength scanning to identify the presence of ferulic acid in the detergent. The detection results were displayed in Figure 4A. It is evident that the ferulic acid–ethanol solution displayed a prominent absorption peak at approximately 320 nm, whereas the detergent solution did not exhibit any noticeable UV absorption characteristics. Consequently, the anhydrous ethanol detergent of the reaction product did not contain free ferulic acid, which further proved that the residual free ferulic acid in the reaction product was washed by anhydrous ethanol.

The hydrolysate of starch ferulate underwent extraction employing ethyl acetate, and ferulic acid was subsequently characterized through UV spectral scanning. As discerned from Figure 4B, the extracted material displayed a reduced absorption peak at 320 nm. This observation could be attributed to the presence of hydrolyzed ferulic acid originating from the reaction product. Importantly, since the product underwent immersion in anhydrous ethanol prior to hydrolysis, the diminished absorption peak in the extract unequivocally indicated the presence of free ferulic acid. This finding indirectly substantiated that ferulic acid and corn starch had undergone synthesis to yield starch ferulate through the process of twin screw extrusion.

### 3.7. FTIR Spectroscopy Analysis

**Figure 5:**
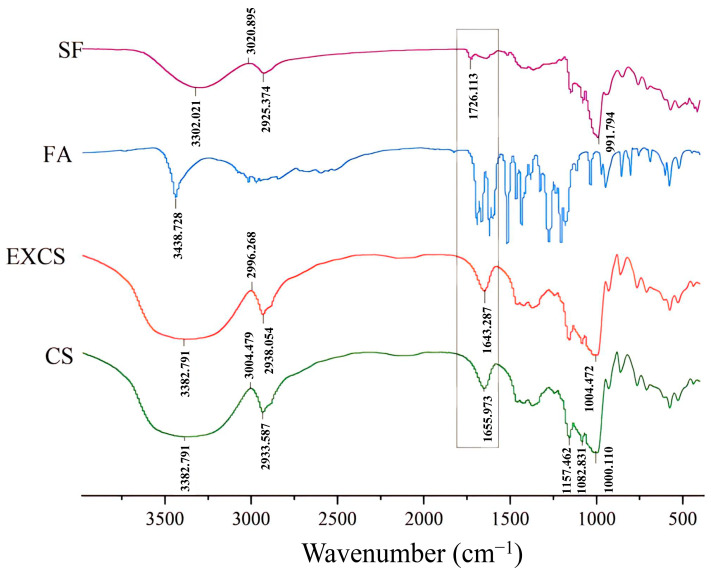
FTIR scan results of different samples.

FTIR was an important technical tool for the structural characterization of products. According to the different vibration frequencies, the absorption peaks of different groups were analyzed to obtain information about the structure and composition of the substance [26]. The infrared spectra of corn starch (CS), extruded corn starch (EXCS), ferulic acid (FA) and starch ferulate (SF) are presented in Figure 5, respectively. For CS, it was evident that a broad peak appeared at 3382.791 cm^−1^, which corresponded to the stretching vibration of starch –OH [27]. The peak of starch C–H was 2933.587 cm^−1^, while the peaks appearing at 1157.462 cm^−1^, 1082.831 cm^−1^, and 1000.110 cm^−1^ corresponded to the C=O peak of starch. For EXCS, the absorption peaks coincide with those of CS, indicating that twin screw extrusion was a physical process that only caused damage to the structure of starch granules and did not involve changes in the moiety. For SF, a new absorption peak appeared at 1726.113 cm^−1^, which corresponded to the ester carbonyl C=O (1660–1850 cm^−1^). The remaining three spectra did not show a similar sharp absorption peak type around 1726.113 cm^−1^, which demonstrated that –OH on the amylose glucose residue (AGU) generated amyl ferulate ester and produced a new functional group (C=O) after twin screw extrusion and enzymatic hydrolysis.

### 3.8. XRD Analysis

**Figure 6:**
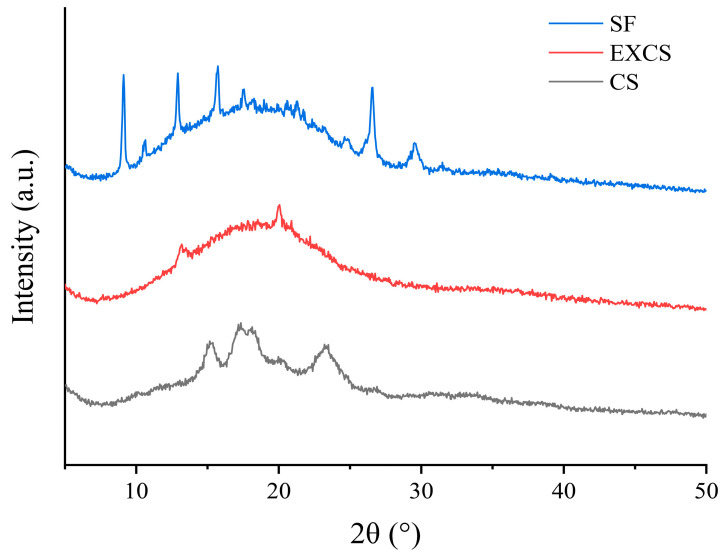
XRD scan results of different samples.

XRD was an effective tool for assessing the crystallinity of crystals. Typically, diffuse peaks indicated that the sample had an amorphous structure, while sharp peaks represented crystalline material [28]. Figure 6 presented the XRD patterns of CS, EXCS and SF, respectively. The diffractograms of CS showed strong diffraction peaks at 15°, 17°, 18° and 23°, indicating that the natural corn starch belongs to the typical A-type crystallization [29]. However, the diffractogram of EXCS tended to be more of a diffuse broad peak, with the sharp features no longer as pronounced. This phenomenon meant that the EXCS might turn out to be an amorphous structure [30]. SEM images confirmed this conjecture. Under the action of extrusion and friction, the crystal structure of the natural starch molecules was destroyed and the lamellar structure replaced the original granular structure. This change provided favorable conditions for the subsequent esterification reaction. So, more and stronger spikes were observed in the diffraction pattern of SF in the range of about 8°–28°. This observation suggested that the esterification reaction between the pasted corn starch and ferulic acid resulted in the emergence of a novel crystal structure. This phenomenon could be attributed to the regeneration and esterification process, wherein the initially disintegrated, short-chained, and straight-chained starch molecules underwent reformation into a double helix structure, subsequently crystallizing [30].

### 3.9. SEM Analysis

**Figure 7:**
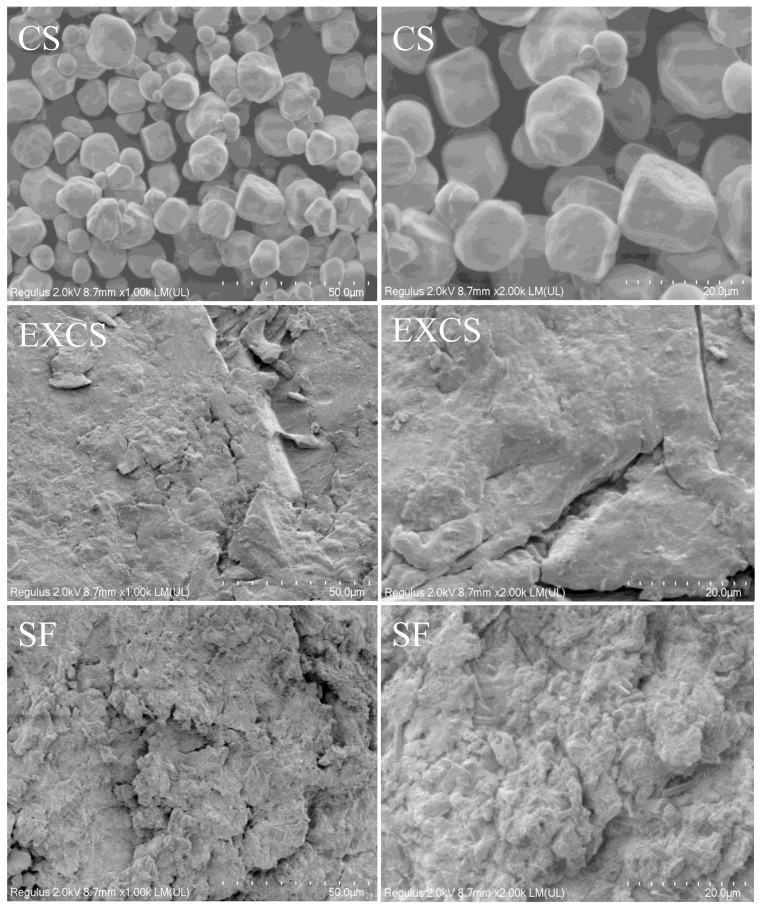
SEM images of different samples at different magnifications.

As depicted in Figure 7, the morphology of the native corn starch granules appeared nearly oval and polygonal, featuring rounded and intact surfaces. Furthermore, the surface displayed various small-sized pores and irregularities. The surface structure of starch granules was destroyed by twin screw extrusion treatment, and the rough surface increased its surface area. Twin screw extrusion treatment exerted forces on starch granules through the shear force and friction of the screw, which damaged the starch crystal structure and impaired the morphology [17]. Furthermore, the surface of the SF exhibited numerous irregular pores, departing from the smooth appearance characteristic of the original corn starch. These surface irregularities exposed a substantial quantity of reactive sites. Under the action of shear force in the twin screw extruder, FS and CS were esterified. The groups inside the starch granules were broken and replaced by acyl groups during the esterification process, which eventually led to a rough granule morphology.

### 3.10. CLSM Analysis

**Figure 8:**
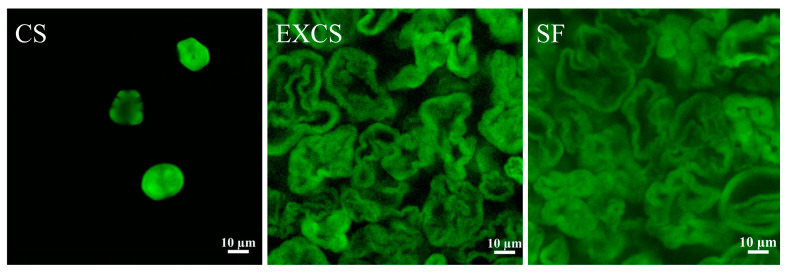
CLSM images of different samples after FITC staining.

CLSM images of different samples were presented in Figure 8. Native corn starch granules exhibited an irregular polygonal or spherical shape with a relatively smooth surface, devoid of noticeable depressions or pore-like structures. However, the surface of the sample modified by twin screw extrusion and esterification was rough and showed a foamy porous structure. Particularly for EXCS, this honeycomb pore structure could expose more hydroxyl sites for contacting and reacting with guest molecules [31]. As for SF, it was suggested that highly substituted starch esters tended to show a honeycomb-like flocculent structure, which was due to esterification reactions on the surface and within the starch.

## 4. Conclusions

In this study, starch ferulate was prepared using the extruded enzymatic complex method and the optimum process parameters and structure were optimized and characterized, respectively. The main conclusions were as follows: (1) The optimal process conditions were 8.2% ferulic acid addition, 66 °C extrusion temperature, and 3.2% lipase addition. The degree of substitution of starch ferulate under this condition was 0.005581. (2) The presence of C=O inside the synthesized starch ferulate meant that the synthesis was successful. Its crystal structure was also reconstructed. (3) The twin screw extrusion technique and the esterification reaction could induce an increase in the specific surface area of starch molecules and the exposure of active sites, which was undoubtedly favorable for the synthesis of starch ferulate. This advancement quietly signifies the immense promise of mechanoenzymatic methods in enhancing the swift and eco-friendly synthesis of starch ferulate, unveiling a fresh perspective in ester research. The insights gleaned from this study extend beyond theory, discreetly guiding the evolution and practical application of modified starch esters.

## Figures and Tables

**Table 1:** Response surface test design and results.

Run	Factor			
	Ferulic Acid (%)	Extrusion Temperature (°C)	Lipase (%)	Degree of Substitution
1	−1	−1	0	0.002916
2	1	−1	0	0.003632
3	−1	1	0	0.003914
4	1	1	0	0.004316
5	−1	0	−1	0.002841
6	1	0	−1	0.003913
7	−1	0	1	0.003965
8	1	0	1	0.003826
9	0	−1	−1	0.002936
10	0	1	−1	0.003644
11	0	−1	1	0.003012
12	0	1	1	0.004728
13	0	0	0	0.005478
14	0	0	0	0.005543
15	0	0	0	0.005314
16	0	0	0	0.005418
17	0	0	0	0.005589

Note: For ferulic acid content, −1, 0 and 1 represent 6%, 8% and 10%, respectively. For extrusion temperature, −1, 0 and 1 represent 60 °C, 65 °C and 70 °C, respectively. For lipase content, −1, 0 and 1 represent 2%, 3% and 4%, respectively.

**Table 2:** Variance analysis table of regression equation.

Factors	df	SS	MS	F-Value	*p*-Value	Significance
A	1	5.258 × 10^−7^	5.258 × 10^−7^	30.28	0.0009	**
B	1	2.107 × 10^−6^	2.107 × 10^−6^	121.34	<0.0001	**
C	1	6.034 × 10^−7^	6.034 × 10^−7^	34.74	0.0006	**
AB	1	2.465 × 10^−8^	2.465 × 10^−8^	1.42	0.2723	/
AC	1	3.666 × 10^−7^	3.666 × 10^−7^	21.11	0.0025	**
BC	1	2.540 × 10^−7^	2.540 × 10^−7^	14.63	0.0065	**
A^2^	1	3.106 × 10^−6^	3.106 × 10^−6^	178.82	<0.0001	**
B^2^	1	3.526 × 10^−6^	3.526 × 10^−6^	203.01	<0.0001	**
C^2^	1	3.989 × 10^−6^	3.989 × 10^−6^	229.68	<0.0001	**
Model	9	1.575 × 10^−5^	1.750 × 10^−6^	100.74	<0.0001	**
Error	7	1.216 × 10^−7^	1.737 × 10^−8^			
Lack of fit	3	7.499 × 10^−8^	2.500 × 10^−8^	2.15	0.2371	/
Pure error	4	4.658 × 10^−8^	1.165 × 10^−8^			
Sum	16	1.587 × 10^−5^				

Note: **, *p* < 0.01

**Table 3:** Reliability analysis of regression model.

Mean	R^2^ (%)	R^2^_Adj_ (%)	COV (%)
4.176 × 10^−3^	99.23	98.25	3.16

## Data Availability

The data presented in this study are available on request from the corresponding author.
